# Eco-Friendly Postharvest Technologies to Preserve or Enhance the Quality and Safety of Fruit and Vegetable Products

**DOI:** 10.3390/foods13121939

**Published:** 2024-06-19

**Authors:** Francisco Artés-Hernández, Lorena Martínez-Zamora, Marina Cano-Lamadrid

**Affiliations:** 1 Postharvest and Refrigeration Group, Department of Agricultural Engineering and Institute of Plant Biotechnology, Universidad Politécnica de Cartagena, 30203 Cartagena, Murcia, Spain; lorena.martinez@upct.es (L.M.-Z.); marina.cano@upct.es (M.C.-L.); 2 Department of Food Technology, Nutrition, and Food Science, Faculty of Veterinary Sciences, University of Murcia, 30071 Espinardo, Murcia, Spain

Fruits and vegetables make up a significant section of the food supply chain and are essential for optimum health and nutrition worldwide [1]. However, their high-quality availability is heavily reliant on good postharvest handling procedures [2,3]. Although significant research has been conducted to preserve the quality and safety of fresh horticultural produce, additional studies on the safety and development of more sustainable technologies are still essential [4].

The aim of this Special Issue was to present recent advances in emerging eco-friendly technologies to maintain or enhance the postharvest quality and safety of fruit and vegetables. It will provide a solid reference for postharvest stakeholders to extend the shelf life, focusing on safety, functional, and sensory characteristics by using environmentally friendly technologies. Relevant researchers around the world have contributed to this collection to improve the knowledge in this field.

Among new eco-friendly technologies, postharvest melatonin applications have recently been investigated due to their great interest. Melatonin is a molecule distributed in nature and plays an important role in animals, humans, and plants. It can delay senescence in horticultural commodities, exert antioxidant effects, regulate growth and development, and facilitate plant adaptation to stress conditions.

Cortés-Montaña et al. (Contribution 1) studied the role of melatonin in some metabolic pathways after its exogenous application (0.1, 0.3, and 0.5 mmol L^−1^) in the sweet cherry cultivar ‘Samba’ for 21 days under controlled cold temperature and humidity. After monitoring the standard quality, respiration rate, sensory quality, phenol concentration, and antioxidant systems (non-enzymatic and enzymatic), their results revealed that a pre-storage application of melatonin, particularly an immersion in 0.5 mmol L^−1^, maintained the enzymatic and non-enzymatic antioxidant systems of ‘Samba’ sweet cherry during storage. This treatment provided firmer cherries without negatively altering skin color and flavor, resulting in improved sensory quality. As a result, using melatonin before storage can be an excellent postharvest technique for preserving the standard, sensory, and bioactive quality of early cherries.

Following this research line, Badiche-El Hilali et al. (Contribution 2) also contributed to this collection with their work about melatonin postharvest treatment in leafy ‘Fino’ lemons. Organic lemons with and without stems and leaves were immersed for 15 and 30 min in 0.01, 0.1, and 1 mM melatonin solutions and stored for 21 days at 2 °C to study the effects on quality. Their obtained results revealed that postharvest applications of melatonin in lemons had a positive consequence on the improvement of the late ripening and in quality by decreasing weight loss, linked to the rate of breathing of the fruit, while increasing firmness or color. The most effective dose was 1 mM melatonin for this outcome. Moreover, lemons that preserved stems and leaves during storage generally showed that they could better maintain quality attributes compared to unleafy lemons, possibly due to greater melatonin absorption through the leaf. Regarding the content of phenolic compounds in both lemon peels and juice, these authors observed the same positive effect on both types of lemons due to the treatment with 1 mM melatonin.

Another interesting research line in postharvest technology is the study of essential oils as natural additives to extend the shelf-life of fresh horticultural products. Pizzo et al. (Contribution 3) investigated the efficacy of essential oil emulsions formulated with oregano (*Origanum vulgare*) and winter savory (*Satureja montana*) at 0.94 and 1.88 μL per mL to reduce the presence of *Escherichia coli* O157:H7 in three varieties of lettuce: romaine, crisphead, and butterhead. The best results were shown by the combination of both essential oils (oregano + winter savory), which was effective in reducing *E. coli* O157:H7 on inoculated lettuce, resulting in reductions of 3.52 (0.94 μL) and 3.41 (1.88 μL) log CFU/g regarding 200 ppm chlorine and 80 ppm peroxyacetic acid. This behavior demonstrates that essential oils can be potential alternatives to chemical sanitizers. Wang et al. (Contribution 4) aimed to improve the postharvest quality of loquat by applying a microemulsion based on an essential oil extracted from *Torreya grandis* cv. Merrillii aril. Such microemulsions were stable for 180 days at room temperature. Moreover, loquat fruit treated with those microemulsions displayed a lower decay index as well as a lower lipid peroxidation after 15 days at 15 °C. The essential oil from *Torreya grandis* cv. Merrillii aril can also be potentially used as a substitute of chemical preservatives to extend the shelf-life of fresh fruit and vegetables.

Knowing the importance of the use of essential oils in the industry as new food preservatives, Gómez-Llorente et al. (Contribution 5) summarized the most relevant studies in the application of antimicrobial agents throughout a deep systematic review about the application of antimicrobials to preserve fruit and vegetables and their derivatives. The use of immobilized antimicrobials in novel dosage forms was investigated by separating two main applications: addition to the food matrix as preservatives and usage during processing as technological aids. After identifying various examples of natural antibacterial chemical immobilization on food-grade supports, the mechanisms of immobilization were thoroughly explored to provide synthesis and characterization guidance for future advances. Finally, Gómez-Llorente et al. (Contribution 5) discussed the novel technology’s contribution to the decarbonization and energy efficiency of the fruit-derived processing sector, as well as the circular economy. In this event, this research highlights how the immobilization of natural antibacterial compounds on food-grade supports has all the characteristics needed to be offered as an eco-friendly postharvest technique capable of protecting fruit-derived foods.

Gómez-Galindo et al. (Contribution 6) contributed to this Special Issue with a work concerning the validation studies performed in two processing lines of shredded iceberg lettuce of a commercial phage biocontrol (PhageGuard ListexTM) for reducing *Listeria monocytogenes*. Their obtained results showed that they optimized the application method, including the device and the process operation steps, reaching the proper concentrations of phages by a fine, mist-like spray with no phage inactivation, and the adequate coverage of the product tested. The post-process treatment with PhageGuard ListexTM did not cause any detrimental impact on the quality, including flavor, texture, browning, spoilage, and visual appearance over the shelf life, as the phage solution was applied as a fine, mist solution.

Lastly, highlighting the need to reduce food losses [5], Martínez-Zamora et al. (Contribution 7) studied the effect of operating conditions on the optimal aqueous ultrasound-assisted extraction of the main phytochemicals in broccoli floret and leaf byproducts. As key biocompounds, glucosinolates, isothiocyanates, and total phenolic content were assayed. Furthermore, two models were considered to elucidate the results obtained in their work: the Peleg model, which is a kinetic model, and an empirical cubic regression model, which were fitted to experimental data over time under fixed conditions of temperature and ratio. As per the main conclusions reached by the authors, the most efficient extraction conditions were at 25 °C, ratio 2:25, and during 15 or 20 min, according to the target phytochemical to extract, being those with the lowest energy consumption.

After wrapping up this Special Issue, we can conclude that postharvest technology is a key strategy to reduce food losses and waste [5], for which it is still an innovative and emerging research line to be developed focusing on eco-friendly technologies.

Finally, the Guest Editors would like to thank all the authors who submitted their papers to this Special Issue and congratulate them for publishing their articles within the valuable journal *Foods*. This would not be possible without the strong support of all devoted reviewers and their constructive comments, for which we are grateful. Furthermore, we would like to thank the MDPI team for their support of this Special Issue.
